# Association of Ego Defense Mechanisms with Electrolyte and Inflammation Marker Levels, Interdialytic Weight Gain, Depression, Alexithymia, and Sleep Disorders in Patients Undergoing Chronic Hemodialysis

**DOI:** 10.3390/jcm13237415

**Published:** 2024-12-05

**Authors:** Đorđe Pojatić, Blaženka Miškić, Ivana Jelinčić, Davorin Pezerović, Dunja Degmečić, Vesna Ćosić

**Affiliations:** 1 Faculty of Dental Medicine and Health Osijek, Josip Juraj Strossmayer University of Osijek, 31000 Osijek, Croatia; 2 Department of Internal Medicine, General County Hospital Vinkovci, 32100 Vinkovci, Croatia; 3 General Hospital “Dr. Josip Benčevic” Slavonski Brod, 35000 Slavonski Brod, Croatia; 4 Faculty of Medicine Osijek, Josip Juraj Strossmayer University of Osijek, 31000 Osijek, Croatia; 5 Department of Psychiatry, University Hospital Centre Osijek, 31000 Osijek, Croatia; 6 Department of Internal Medicine, University Hospital Centre Osijek, 31000 Osijek, Croatia; 7 Polyclinic Ćosić, 35000 Slavonski Brod, Croatia

**Keywords:** alexithymia, defense mechanisms, depression, leukocytes, sleep quality, phosphorus

## Abstract

**Background/Objectives**: Ego defense mechanisms are subconscious processes that help individuals cope with stressors from both external and internal realities. They are divided into three levels based on their adaptive function. Patients undergoing chronic hemodialysis are those who have been treated with this method for longer than three months. Only a few studies have examined the defense mechanisms in hemodialysis patients. Our study aimed to examine the association between ego defense mechanisms and alexithymia, depression, and sleep disorders, as well as clinical and biochemical variables, in a group of 170 hemodialysis patients. **Methods**: We used the Defense Style Questionnaire-40, the Toronto Alexithymia Scale-26, the Pittsburgh Sleep Quality Index, and the Hamilton Depression Inventory as our analyses methods. Clinical and biochemical variables, along with interdialytic weight gain, were measured before the hemodialysis session. **Results:** There was a positive correlation between the affect displacement and dissociation with leukocyte levels (Spearman’s rho = 0.192, *p* = 0.02; rho = 0.165, *p* = 0.04), and between autistic fantasy and phosphorus levels (rho = −0.163, *p* = 0.04). Depressive HD patients had higher levels of somatization, affect displacement, and splitting compared to the HD patients without depression (Man–Whitney U test, *p* = 0.005, *p* = 0.022, *p* = 0.045). There were higher levels of immature defense mechanisms in the group of patients with alexithymia than in the group without alexithymia (Mann–Whitney U test, *p* < 0.001). **Conclusions**: The immature defense mechanisms were our research model’s strongest predictive factor of alexithymia (OR = 1.87, 95% CI 1.27 to 2.75).

## 1. Introduction

Since 1966, the treatment of end-stage kidney disease (ESKD) became possible when Dr. Scribner developed hemodialysis [1]. Hemodialysis involves replacing kidney function with a device that facilitates the diffusion and ultrafiltration of particles from the patient’s blood into an extracorporeal solution called dialysate. The treatment requires several procedures per week, each lasting 3 to 5 h, depending on the patient’s condition [2]. Although hemodialysis treatment has extended the lives of patients with ESKD, it has raised numerous other issues related to mental health and quality of life in this vulnerable population. Due to the pathophysiological consequences of reduced kidney function, such as the accumulation of uremic toxins, secondary anemia, and secondary hyperparathyroidism, patients with ESKD suffer from numerous somatic problems, including dermatological conditions, susceptibility to pathological fractures, frequent infections, and others [3]. Consequently, the restrictions imposed by life on renal replacement therapy—such as fluid and dietary restrictions, constant witnessing of complications experienced by other patients, the necessity of taking a large number of medications, and other comorbidities—leave hemodialysis patients (HD patients) constantly exposed to frustrations due to this treatment regimen [4].

In states of high exposure to stressors, ego defenses become more pronounced. This concept was developed by Anna Freud between 1936 and 1946 and involves the subconscious mechanisms aimed at reducing anxiety when a person comes into conflict with their external or internal realities, which cause the aforementioned frustrations [5]. Based on the level of reality insight they allow the individual, defenses are divided into three levels: immature, neurotic, and mature. Mature ego defenses, such as humor, not only allow the individual to release anxiety caused by the stressor but also offer the possibility of gaining insight into reality, giving the person a chance to consciously change the source of frustration and, in that sense, reality itself. Some of the mature ego defenses, which are hierarchically the highest, enable progress in most areas of life. For example, sublimation allows frustration to be used as motivation for progress in other fields [6]. Neurotic defenses provide partial insight into reality, but they put the individual at a disadvantage in relationships with others, as the person gives up on their goals and leaves them to others. For instance, in pseudo-altruism, a person who is hindered by a real frustration abandons their goal and tries to help others instead [7] thus missing out on feelings of satisfaction. At the bottom of this hierarchical chain are immature defenses, which represent the lowest level of coping with reality because they provide no insight into the source of frustration, leaving the individual with little ability to influence it. The concept of ego defenses assumes that defenses are always more pronounced when frustration is present, that they have adaptive potential, meaning they maintain the person’s psychological stability, and that they change with age or with the duration of exposure to the stressors [8]. Some studies have shown a higher prevalence of certain immature ego defenses, such as denial, projection, and somatization, in HD patients compared to the general population [9].

Given that immature ego defenses in the general population are associated with overeating, alcohol consumption, and a sedentary lifestyle, it is interesting to explore whether there is a connection with certain clinical and laboratory variables in HD patients [10]. Interdialytic weight gain, or the change in body weight between two dialysis sessions, is highly dependent on the amount of food and water consumed, and it may be linked to an immature response to constant frustrations. Additionally, pre-dialysis levels of phosphorus, and more importantly potassium, are heavily influenced by the consumption of certain types of food and their quantities [11]. Thus, mental defense mechanisms in the context of conflict with reality may determine the clinical condition of the individual. Furthermore, higher levels of use of hierarchically higher defenses, such as neurotic and mature defenses, may positively affect the mentioned variables. It has also not been examined whether there is a correlation between specific neurotic and immature ego defenses and inflammatory markers in these patients, considering that defenses involving aggression could lead to a stress response in the body and thus elevate leukocyte and C-reactive protein levels.

High levels of use of immature and neurotic ego defenses are associated with various psychiatric disorders, including depression. The prevalence of depression in HD patient samples reaches up to 45%, which is almost 4 to 5 times higher than the prevalence of this disorder in the general population [12]. According to the DSM-5 classification, depressive disorder is defined as a state of loss of interest or pleasure in everyday activities lasting longer than two weeks, accompanied by at least four of the following factors: weight loss or loss of appetite, insomnia or excessive sleepiness, the presence of suicidal thoughts, feelings of helplessness or persistent guilt, psychomotor agitation or retardation, and a constant feeling of fatigue or loss of concentration [13]. In every population, depressive disorder is associated with older age, the number of comorbidities, female gender, and the loss of a partner. In HD patients, there are additional pathophysiological mechanisms and lifestyle factors that can contribute to the development of depression. Among the many frustrations faced by HD patients, ego defenses simply must become more pronounced in order for the ego to cope with the extreme amount of stress, and the dominant type of defense may be linked to the preservation of mental health and treatment outcomes [14]. In previous studies on general population samples, depressive disorder has been associated with pronounced immature ego defenses, while in psychotherapeutic treatment, the level of immature defenses decreases as depression levels in depressive individuals decrease [15]. Immature ego defenses, as mentioned earlier, do not allow cognitive insight into the stressor, and thus prevent conflict resolution. Additionally, immature defenses such as somatization can further complicate diagnostic evaluations in HD patients, who already suffer from numerous potential ailments, and can mask depression. Therefore, one of the aims of this study is to measure the level of individual and overall ego defenses and compare them between depressive HD patients and those without depression.

Alexithymia, which is defined as a deficit in recognizing emotions, is a factor that predisposes individuals in various clinical samples to the development of depression. Some recognize it as a mental mechanism similar to immature ego defenses [16]. Alexithymia is defined as the inability to recognize one’s own emotions or describe emotions to others, and a way of thinking that is driven solely by external factors rather than feelings [17]. Some researchers have defined alexithymia as a primitive defense mechanism because it does not allow or reduce the experience of emotions thereby preventing the frustration or dissatisfaction that arises from them. Many researchers debate whether alexithymia is truly a separate mental mechanism in the form of a cognitive deficit or if it is a primitive ego defense mechanism, given that they serve a similar function. Supporting the idea of an organic origin for alexithymia is the fact that levels of alexithymia are higher in HD patients with more severe comorbidities, those who have been on dialysis longer, or those who are older [18]. In previous studies on clinical samples, levels of alexithymia were associated with immature ego defenses and higher levels of depression, which has not yet been investigated in the clinical sample of HD patients [19,20].

Sleep quality has often been studied in the population of HD patients, and most studies have linked reduced sleep quality with older age, the number of comorbidities, and psychological disorders [21]. Among other things, as in the general population, there is a positive correlation between depressive disorder and reduced sleep quality in HD patients. Furthermore, reduced sleep quality leads to higher levels of daytime sleepiness and is a negative prognostic factor for low quality of life and survival. According to previous studies, there is a vicious cycle of negative feedback between sleep disorders and depression, where one reinforces the other. In this “vicious cycle”, the way of coping with everyday stressors to which these severely somatic patients are exposed can further modulate the relationship between depression and sleep quality [22]. We can say that alexithymia, as a primitive defense mechanism, is a factor that reduces HD patients’ adaptive capacity to everyday stressors and thus has a negative effect. It remains unclear whether other unconscious mechanisms, such as ego defenses, are also associated with better or worse adaptation to daily stressors and whether they are linked to the mental health of HD patients.

## 2. Materials and Methods

A cross-sectional multicenter study was conducted, approved by the relevant ethics committees. It was conducted at the Nephrology Department of University Hospital Centre Osijek (authorization number R2-1160/2020), at the Dialysis Unit of the Healthcare Center Županja (authorization number 381-12-19-01-1), and at the General County Hospital Vinkovci (authorization number 01-9206/3/19).

### 2.1. Subjects

The sample of subjects was selected by using the “simple random sampling” method, where a research assistant drew papers from a drum. The papers contained the date of each patient’s first hemodialysis session, as no two patients in the entire study had started dialysis on the same day. The date of the first hemodialysis session was converted into an eight-digit code, under which all other medical data of the patients could be accessed anonymously. This code was also used to label the sampled blood specimens of the subjects. All subjects signed an informed consent form for participation after being informed about all the details of the study. They completed questionnaires for the assessment of ego defenses, alexithymia, and sleep disorders, and were interviewed about the severity of depressive symptoms. The study began in February 2020 and lasted for one year.

### 2.2. Selection of Subjects for Participation in Study

The inclusion criteria were the following: hemodialysis patients of both sexes, adults, and who gave consent for the study and had been treated with hemodialysis twice a week for at least three months.

The exclusion criteria were the following: inadequately completed questionnaires, withdrawal of consent to participate in the study, patients with previously diagnosed psychiatric disorders, and patients with acute somatic events, including the period of three months before the start of the study.

### 2.3. Variables and Methods Used in the Study

The total duration of hemodialysis treatment, the number of dialysis sessions per week, and the duration of individual hemodialysis sessions were recorded. Interdialytic weight gain (IDWG) was recorded according to the following formula: body weight at the start of hemodialysis treatment—body weight at the end of the previous hemodialysis session, divided by the number of days between the two hemodialysis sessions [23]. During the week in which the subjects were interviewed, laboratory values for leukocytes, CRP, and electrolytes, phosphorus and potassium were measured before each individual hemodialysis session [24]. The subjects completed the Defense Mechanism Questionnaire (DSQ-40), the Toronto Alexithymia Scale (TAS-26), the Epworth Sleepiness Scale (ESS), and the Pittsburgh Sleep Quality Index (PSQI), while depressive symptoms were assessed by a trained professional using the Hamilton Depression Rating Scale (HDRS-20). For the assessment of ego defenses, a self-report scale was used: the Defense Style Questionnaire (DSQ-40). The DSQ-40 measures four mature, four neurotic, and twelve immature ego defenses. Each ego defense is represented in the questionnaire by 2 items, and responses are given on a 9-point Likert scale according to the subject’s agreement with the statement (1—strongly agree; 9—strongly disagree). The score for each ego defense is obtained by scoring the items in a positive direction and calculating their arithmetic mean. The score for each subscale is obtained by calculating the arithmetic mean of the ego defenses represented in that subscale.

The questionnaire was validated in Croatian and shows internal reliability (Cronbach a = 0.89) [25].

The TAS-26 scale was used to measure alexithymia. The scale consists of 26 questions grouped into 4 subscales that measure difficulties in identifying feelings, difficulties in describing one’s own feelings, reduced ability for fantasy, and externally oriented thinking. Based on the results of the TAS-26 questionnaire, the subjects were divided into the following groups: those without alexithymia, those with a moderate level of alexithymia, and those with pronounced alexithymia. The groups with moderately pronounced alexithymia and pronounced alexithymia were then combined into a group of alexithymic patients. The internal reliability of the overall scale was satisfactory (Cronbach a = 0.71). Subjects respond to the questions on a 5-point Likert scale according to their agreement with the statements (1—strongly agree; 5—strongly disagree). Subjects who score between 63 and 72 points are moderately alexithymic, while those who score 62 or fewer points on the one side, or 74 or more points on the other side are either without alexithymia or alexithymic, respectively [26].

The Hamilton Depression Rating Scale, which measures depressive symptoms over the past week, was used to evaluate symptoms of depression. The patients were evaluated by a psychiatrist using 17 questions, through observation and asking questions. Based on the scale results, patients with 8 or more points were classified as depressed, while those with 7 points or fewer were classified as non-depressed. The internal reliability of the questionnaire is good (Cronbach a = 0.90) [27,28].

Sleep quality was assessed using the PSQI scale, which consists of 19 questions that evaluate the subjective experience of sleep quality. The maximum score on the questionnaire is 21, with higher scores indicating lower sleep quality. Subjects who scored 5 or more points were categorized as “poor sleepers”. The questionnaire has been frequently used in clinical samples of hemodialysis patients, where it has been validated and has shown good psychometric properties (Cronbach a = 0.83) [28].

Daytime sleepiness was assessed using the self-reported Epworth Sleepiness Scale (ESS), which consists of 8 questions. Scoring is carried out on a Likert scale from 0 to 3 points, where higher scores for each question indicate higher levels of daytime sleepiness. Patients who scored 10 or more points on the questionnaire were grouped into the category of patients with increased daytime sleepiness. The questionnaire shows high internal reliability (Cronbach a = 0.84) [29].

### 2.4. Statistical Methods

Categorical data are presented as absolute and relative frequencies, whereas numerical data that follow a normal distribution are presented as the arithmetic mean and standard deviation, and data that follow a non-parametric distribution are presented as the median and interquartile range. The relationship between numerical variables was examined using Spearman’s correlation coefficient, ρ (rho). For data that follow a non-parametric distribution, the Mann–Whitney U test and the Kruskal–Wallis test were used.

The Mann–Whitney U test (with the Hodges–Lehmann median difference) was used to assess the differences between the numerical variables in the two independent groups. For testing the differences between the two groups of data that follow a normal distribution, the independent *t*-test was used.

Logistic regression was used to assess the impact of the following variables: leukocyte levels, phosphorus levels, depression levels, sleep quality, and immature and neurotic defense mechanisms on the occurrence of alexithymia. All *p*-values are presented as two-tailed *p*-values, with the significance level set at *p* = 0.05. Data analysis was performed using SPSS (IBM Corp. Released 2015. IBM SPSS Statistics for Windows, Version 23.0 Armonk, NY, USA, IBM Corp.) [30].

## 3. Results

### 3.1. The Basic Characteristics of the Subjects

The study was conducted on 170 subjects, 99 (58.2%) of whom were male, while 71 (41.8%) were female. The median age of all subjects was 67 (interquartile range 58–74 years of age), ranging from 20 to 87 years of age. The median duration of dialysis was two years (ranging from 90 days to 35 years). Of the total number, 145 subjects (85.3%) underwent dialysis 3 times a week, while 23 patients (33.5%) underwent dialysis 2 times a week, and only 2 patients (1.2%) underwent dialysis 4 times a week. The majority of patients, a total of 124 (79%), had 4 h dialysis sessions. Eleven patients (7%) underwent dialysis for 3.5 h, 4 patients (2.5%) for 3 h, while the rest underwent dialysis for more than 4 h. The median interdialytic weight gain was 0.3 kg (interquartile range 0.01–1.2 kg), ranging from 0 kg to 2.25 kg. The values of the biochemical variables and ego defenses are shown in Table 1 and Table 2.

### 3.2. Correlation of Total and Individual Ego Defenses with Interdialytic Weight Gain, Inflammatory Variables (Leukocytes and CRP), and Electrolytes (Pre-Dialysis Potassium and Phosphorus Levels)

We assessed the correlation of the total and individual ego defenses with the inflammatory variables and electrolytes using Spearman’s correlation coefficient. Only significant results are highlighted. There is a significant positive correlation between interdialytic weight gain and levels of humor (rho = 0.231, *p* = 0.09) and sublimation (rho = 0.202, *p* = 0.023). There is also a mildly positive correlation between interdialytic weight gain and the level of pseudo-altruism (rho = 0.210, *p* = 0.018).

There is a correlation between leukocyte levels and certain individual immature ego defenses, such as dissociation (rho = 0.227, *p* = 0.007), splitting (rho = 0.230, *p* = 0.006), and affect displacement (rho = 0.211, *p* = 0.013).

The pre-dialysis phosphorus levels are inversely correlated with levels of autistic fantasy (rho = −0–203, *p* = 0.016) and denial (rho = −0.199, *p* = 0.018).

### 3.3. Differences in Ego Defenses by Groups Based on Presence of Alexithymia

There are no differences in the level of mature ego defenses between groups based on the presence of alexithymia (independent *t*-test; F = 2.873, *p* = 0.763). The alexithymic HD patients show significantly higher levels of neurotic ego defenses (independent *t*-test; F = 0.341; *p* = 0.017). Within the neurotic ego defenses subscale, repression (Mann–Whitney U test; *p* = 0.033) and idealization (Mann–Whitney U test; *p* = 0.012) are significantly more pronounced in alexithymic patients. Immature ego defenses are significantly more pronounced in the group of HD patients with pronounced alexithymia (independent *t*-test; F = 0.383; *p* = 0.002) (Table 3).

### 3.4. Differences in Ego Defense Levels Based on Presence of Depressive Symptoms

There are no differences in the levels of mature ego defenses between the group of patients with depression and those without depression (independent *t*-test; F = 0.417; *p* = 0.459). There are no differences in the levels of neurotic defenses between the groups of patients based on the presence of depression (independent *t*-test; F = 0.107; *p* = 0.448). There are no differences in the levels of total immature defenses between the groups of subjects based on the degree of depression (Mann–Whitney U test, *p* = 0.086), while the levels of somatization (Mann–Whitney U test, *p* = 0.005), affect displacement (Mann–Whitney U test, *p* = 0.022), and splitting (Mann–Whitney U test, *p* = 0.045) are higher in the groups of depressive patients (Table 4).

### 3.5. Defense Values Based on Sleep Quality and Daytime Sleepiness

There are no differences in the levels of total mature defenses (independent *t*-test; F = 0.003, *p* = 0.408), immature defenses (independent *t*-test; F = 0.01; *p* = 0.558), or neurotic defenses (independent *t*-test; F = 0.539; *p* = 0.809) between the groups of patients with good and reduced sleep quality. There are no differences in the levels of mature ego defenses (independent *t*-test; F = 0.178; *p* = 0.908) or neurotic ego defenses (independent *t*-test; F = 1.019; *p* = 0.677) between the groups of patients based on the level of daytime sleepiness. The levels of immature ego defenses are borderline significantly higher in the group of patients with increased daytime sleepiness (Mann–Whitney U test; *p* = 0.057). Among individual immature ego defenses, only the levels of affect displacement are higher in the group of patients with pronounced daytime sleepiness (Mann–Whitney U test; *p* = 0.043) compared to those without pronounced daytime sleepiness, while there are no differences in the levels of other immature defenses.

### 3.6. Quality of Dialysis and Correlation with Psychometric Characteristics

We examined whether there is a correlation between ego defenses and quality of dialysis measured by the Kt/V variable.

There is no significant correlation between mature (rho = 0.064, *p* = 0.697), neurotic (rho = −0.025, *p* = 0.878), and immature ego defenses (rho= −0.960, *p* = 0.556) and dialysis quality in our sample assessed by Spearman’s correlation coefficient.

As shown in our results in Table 5, there are no statistical differences in the quality of dialysis of HD patients according to the presence of alexithymia, daytime sleepiness, sleep quality, and depression. 

### 3.7. Predictors of Alexithymia in HD Patients

To examine which predictive factors influence the likelihood of subjects having alexithymia, we considered eleven independent variables (sex, age, duration of HD, CRP, uric acid, leukocytes, phosphorus, Hamilton Depression Rating Scale, sleep quality, immature defense mechanisms, and neurotic defense mechanisms). The significance of individual predictive factors in predicting alexithymia is presented using bivariate regression.

As a model, we observed the factors that were significant (leukocytes, pre-dialysis phosphorus, Hamilton Depression Rating Scale, sleep quality, and immature and neurotic defense mechanisms). The model is statistically significant overall, χ^2^ = 21.4, *p* < 0.001, indicating that it can distinguish subjects based on the clinical presentation of alexithymia. The model as a whole explains between 16.9% (according to Cox and Snell) and 24.5% (according to Negelkerke) of the variance in the presence of alexithymia and correctly classifies 73.9% of cases.

Only two independent predictive factors made a uniquely significant contribution to the model (immature defense mechanisms and the Hamilton Depression Rating Scale). The stronger predictive factor was immature defense mechanism (OR = 1.87) (Table 6).

## 4. Discussion

Although the initial assumption was that, like the general population, HD patients would be prone to consuming larger quantities of food as a function of immature ego defenses (as is the case in the general population), this correlation was not significant [10]. Contrary to this assumption, a correlation was found between humor and sublimation with higher interdialytic weight gain; moreover, even the neurotic defense of pseudo-altruism is associated with higher interdialytic weight gain. Thus, the use of mature defenses like humor and sublimation could be an indirect indicator of a higher biological reserve. Adequate coping with external frustrations in the form of hierarchically higher defenses is associated with preserved appetite in HD patients and likely with better psychophysical condition, which needs to be confirmed in longitudinal studies.

The correlation between immature ego defenses such as splitting, affect displacement, and dissociation with leukocytosis in HD patients should be interpreted in relation to the levels of negative affect that these responses provoke in the individual. For example, affect displacement often causes an individual to display aggressive behavior toward an object different from the one that originally caused the negative affect thus activating the sympathetic nervous system response [31]. This leads to the release of catecholamines and stress-induced mobilization of white blood cells, which manifests as leukocytosis and occurs merely as a consequence of acute reaction to stress in HD patients.

Our research results indicate that levels of immature ego defenses, denial, and autistic fantasy are associated with lower pre-dialysis phosphorus levels in HD patients. Similar findings were observed in our previous study, showing an inverse relationship between alexithymia levels and pre-dialysis phosphorus, interpreted as alexithymic HD patients eating less due to lower biological reserves linked to reduced cognitive capacity. Given the significant overlap between the concept of alexithymia and immature ego defenses, it is possible that similar mechanisms are at play. High levels of denial and autistic fantasy in individuals with low phosphorus levels may reflect the consequences of reduced cognitive capacity [32,33,34].

Alexithymia as a personality trait can be considered relatively stable throughout life [35]. A meta-analysis conducted in 2013 found several neural correlates associated with alexithymia, as the study concluded that the amygdala shows reduced activity when processing negative emotional stimuli in individuals with alexithymia, resulting in weaker emotional performance [36]. A study conducted in 2023 shows that the prevalence of alexithymia in the population of chronic kidney disease patients is higher than in the control group of healthy individuals [37]. In alexithymic HD patients, specific defense mechanisms are present that help cope with accumulated stress, chronic illness, and uncertain health outcomes. This also includes the findings in this study of higher levels of neurotic and immature ego defenses. Our results showed that, among neurotic ego defenses, HD patients most frequently use repression, which helps the ego tolerate intense, threatening anxiety that would arise from the insight into unpleasant reality. Alexithymic HD patients in the examined sample idealize significantly more, which makes it easier for them to psychologically cope with chronic stress and the feeling of losing control over their lives. By using idealization, they reduce tension, believing that the healthcare staff will make the right decisions.

Immature ego defenses are characteristic of individuals who have difficulty with emotional regulation. In our study, the group of HD patients with pronounced alexithymia scored significantly higher on the scales of immature ego defenses. This can be explained by the assumption that due to the alexithymic pattern of thinking, where individuals remain unaware of their emotions, they resort to pushing frustrations into the subconscious through immature ego defenses. [20]. A study conducted on hemodialysis patients in Italy found a wide range of defense mechanisms in this population (repression, splitting, passive-aggressive behavior, reaction formation, and autistic fantasy) [38]. This suggests that it is necessary to adapt the therapeutic approach to include various techniques aimed at improving the ability to regulate emotions, especially in HD patients with preserved cognitive capacity and adequate personality structure. A broader understanding of how defense mechanisms affect the emotional state of the patient is needed, as they can either contribute to or hinder the process of specific and long-term treatment. The development of cognitive skills required for identifying and expressing emotions is a potentially useful tool in patient care in the long run [19].

In HD patients with depressive symptoms, the levels of somatization, affect displacement, and splitting are elevated compared to non-depressive HD patients, as indicated by the results. This can be explained by various psychopathological and psychodynamic mechanisms. Due to constant physical stress and discomfort, patients are prone to somatization, where emotional stress is channeled through somatic symptoms, providing compensation when verbal communication is inadequate or impossible. Depressive HD patients rely significantly more on splitting, which under conditions of psychophysical stress leads to active aggression and conflicts with family members and others in the environment, triggering even higher levels of negative affect and obscuring the true source of frustrations. The use of splitting in depressive HD patients involves alternating feelings of relief following individual hemodialysis sessions and despair over the lifelong dependence on this method. In line with the results of this study, a study conducted by Carvahlo et al. concluded that immature defense mechanisms were observed in individuals with a depressive temperament [39], while a study by Preece et al. concluded that all aspects of alexithymia were significantly associated with symptoms of depression, anxiety, and stress [40]. Several meta-analytical studies and reviews report a correlation between alexithymia and depression and anxiety [41,42,43,44]. The clinical benefit of our study lies in the clearly defined need for a periodic assessment of the degree of depressive symptoms and ego defense levels in HD patients, as these factors are key determinants of their quality of life, overall morbidity, mortality, and treatment compliance. Given the frequent use of anxiolytics in this population, our study provides insights into the degree of depressive symptomatology and strategies of coping with frustrations, further justifying the use of supportive psychotherapy and antidepressants to manage symptoms, tailored to each individual case.

There are several possible reasons why we associate increased daytime sleepiness with higher levels of immature defense mechanisms. Higher levels of daytime sleepiness can reduce cognitive function and an individual’s ability to adequately solve problems. This can lead to an increased use of immature defense. Sleepiness affects emotional regulation and mood control, potentially raising stress levels, making individuals more likely to resort to using immature defense mechanisms. [45]. A 2006 study suggests that immature defense mechanisms are also often present in individuals with depression and anxiety, conditions that may be linked to increased daytime sleepiness [46].

Depression and alexithymia share common as well as specific characteristics. Difficulties in emotional recognition and expression, a reduced ability to cope with stress, and diminished social interactions are just some of the commonalities. Accordingly, the predictors of alexithymia are depression and immature ego defenses, with a note that immature defenses have a higher predictive potential for alexithymia. Although depressive states, accompanied by reduced cognitive capacity due to a lack of neurotransmitters, support alexithymic thinking, immature ego defenses are stronger predictors of alexithymia. Why this is the case, whether these two entities are actually the same, and whether the function of alexithymia is to reduce frustration in confrontation with emotionally unacceptable factors—similar to the function of immature defenses—or whether the nature of alexithymia lies in the biological organization of neurons and cognitive deficits remains to be determined by future studies.

## 5. Conclusions

Through a cross-sectional study, we have established associations between variables of mental and somatic health in HD patients. There is a correlation between serum phosphorus levels and leukocyte count with unfavorable mental processing of frustrations, as well as a correlation between interdialytic weight gain and more mature ego defenses among HD patients in dealing with daily frustrations. Through the assessment of depression symptoms, ego defenses, daytime sleepiness, and alexithymia, we have highlighted the link between immature reality processing in this population and mental health. These variables showed no correlation with dialysis quality, indicating a need for further research to better understand their relationship with limitations due to ESKD and hemodialysis treatment. Given that alexithymia and immature ego defenses were the most significant predictors in our model of studied variables, further research is necessary to evaluate the impact of psychotherapeutic methods and medication therapy aimed at improving the mental health of this fragile population.

## Figures and Tables

**Table 1:** Biochemical variable values.

	Median (Interquartile Range)	Minimum–Maximum
CRP	4.68 (1.89–10–68)	0.2–130
Leukocytes	6.4 (5.2–8.1)	1.3–14.3
Pre-dialysis potassium	4.9 (4.5–5.4)	3.2–5.9
Pre-dialysis phosphorus	1.54 (1.19–1.86)	0.5–3.6

**Table 2:** Subscale values of DSQ questionnaire in HD patients.

	Median (Interquartile Range)	Minimum–Maximum
Mature defenses	6 (4.75–7.12)	1–9
Immature defenses	4.12 (3.12–5.25)	1–8.79
Neurotic defenses	5 (4.12–6.37)	1–9

**Table 3:** Differences between individual immature ego defenses based on presence of alexithymia.

	Median (Interquartile Range)		Difference	95% Confidence Interval	*p* *
	Subjects Without Alexithymia	Alexithymic Subjects			
**Isolation of affect**	3 (1–5)	5 (3–6.5)	−1.5%	−2.5–−0.5%	**0.004**
**Devaluation**	3.5 (1.625–5)	5 (3–5.75)	−1%	−2–0%	**0.005**
**Splitting**	3 (1–4.875)	5 (3–6.5)	−1.5%	−2.5–−1%	**0.000**
**Affect displacement**	1.5 (1–3.5)	4 (1.5–5)	−1%	−2–0%	**0.002**
**Autistic fantasy**	3 (1–4.875)	4 (1.5–5)	−1%	−2–0%	**0.021**
**Somatization**	1.5 (1–4.5)	4 (1.75–5.25)	−1%	−2–0%	**0.005**
**Projection**	2.5 (1–4.875)	4 (2–5)	−0.5%	−1.5–0%	**0.028**
Passive aggression	3.5 (1–5)	5 (3–5)	−0.5%	−1.5–0%	0.062
Acting out	4.25 (1.12–6.87)	5 (3.5–7)	−1%	−2–0%	0.051
Rationalization	5.75 (4.62–8.37)	6.5 (4–8.5)	0%	−1–1%	0.836
Dissociation	3.25 (1–5)	4 (2–5)	−0.5%	−1.5–0%	0.156
Denial	4 (2.5–6)	5 (3–6.5)	−0.5%	−1.5–0%	0.179

* Mann–Whitney U test; % Hodges–Lehmann estimator.

**Table 4:** Values of individual immature defenses based on presence of depression.

	Median (Interquartile Range)		Difference %	95% Confidence Interval	*p* *
	Subjects Without Depressive Symptoms	Subjects With Depressive Symptoms			
**Somatization**	2 (1–5)	4.5 (2–6)	−1.5%	−25–0%	**0.005**
**Affect displacement**	2 (1–4.25)	4 (1.5–5)	−1%	−2–0%	**0.022**
**Splitting**	4.5 (1.5–5)	5 (3.25–7)	−1%	−2–0%	**0.045**

* Mann–Whitney U test; % Hodges–Lehmann estimator.

**Table 5:** Values of dialysis quality (Kt/V) according to alexithymia, sleep quality, daytime sleepiness, and depression levels.

	Arithmetic Mean (Standard Deviation)		Difference ^%^	95% Confidence Interval	*p* *
	Expressed Symptoms	Without Symptoms			
Alexithymia	1.53 (0.39)	1.72 (0.33)	0.19%	−0.05–1.43%	0.125
Sleep quality	1.58 (0.37)	1.65 (0.42)	0.066%	−0.24–0.37%	0.651
Daytime sleepiness	1.52 (0.27)	1.62 (0.40)	0.091%	−0.17–0.36%	0.468
Depression	1.65 (0.38)	1.47 (0.44)	−0.17%	−0.50–0.14%	0.268

* Independent *t*-test; % Levene’s test for equality of variances.

**Table 6:** Prediction of probability of alexithymia in subjects (bivariate and multivariate regression analysis).

Predictive Factors	ß	Standard Error	Wald	Odds Ratio (OR)	95% Confidence Interval	*p*
**Bivariate regression**						
Sex	0.04	0.37	0.01	1.04	0.51–2.13%	0.91
Age	0.01	0.01	1.22	1.01	0.98–1.04%	0.27
Duration of HD	0.05	0.05	1.15	1.05	0.95–1.16%	0.28
CRP	0.003	0.01	0.06	1.003	0.97–1.03%	0.81
**Leukocytes**	0.23	0.09	6.02	1.26	1.05–1.51%	**0.01**
**Phosphorus**	−0.88	0.36	0.09	0.41	0.21–0.83%	**0.01**
**Hamilton Depression Rating Scale**	0.11	0.04	7.23	1.12	1.03–1.21%	**0.006**
**PSQI total**	0.09	0.05	4.56	1.10	1.01–1.21%	**0.03**
**Sleep quality (poor)**	0.42	0.39	1.19	1.53	0.72–3.26%	0.27
**Immature defense mechanisms**	0.53	0.15	11.9	1.69	1.26–2.29%	**0.001**
**Neurotic defense mechanisms**	0.24	0.12	4.04	1.27	1.01–1.59%	**0.04**
**Multivariate regression (Stepwise method)**						
**Depression (Hamilton Scale)**	0.09	0.04	4.20	1.09	1.01–1.19%	**0.04**
**Immature defense mechanisms**	0.63	0.19	10.1	1.87	1.27–2.75%	**0.002**
**Constant**	−2.2	0.81	7.5			**0.006**

ß—regression coefficient.

## Data Availability

The data of the patients included in this study are available from the lead author.
